# Toward *Operando* Characterization of Interphases in Batteries

**DOI:** 10.1021/acsmaterialslett.3c00207

**Published:** 2023-08-10

**Authors:** Julia Maibach, Josef Rizell, Aleksandar Matic, Nataliia Mozhzhukhina

**Affiliations:** Department of Physics, Chalmers University of Technology, SE 412 96, Göteborg, Sweden

## Abstract

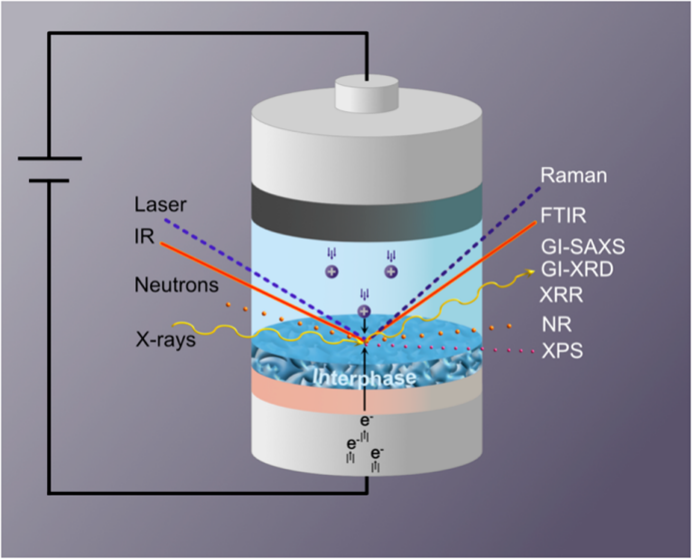

Electrode/electrolyte interfaces are the most important and least understood components of Li-ion and next-generation batteries. An improved understanding of interphases in batteries will undoubtedly lead to breakthroughs in the field. Traditionally, evaluating those interphases involves using *ex situ* surface sensitive and/or imaging techniques. Due to their very dynamic and reactive nature, *ex situ* sample manipulation is undesirable. From this point of view, *operando* surface sensitive techniques represent a major opportunity to push boundaries in battery development. While numerous bulk spectroscopic, scattering, and imaging techniques are well established and widely used, surface sensitive *operando* techniques remain challenging and, to a larger extent, restricted to the model systems. Here, we give a perspective on techniques with the potential to characterize solid/liquid interfaces in both model and realistic battery configurations. The focus is on techniques that provide chemical and structural information at length and time scales relevant for the solid electrolyte interphase (SEI) formation and evolution, while also probing representative electrode areas. We highlight the following techniques: vibrational spectroscopy, X-ray photoelectron spectroscopy (XPS), neutron and X-ray reflectometry, and grazing incidence scattering techniques. Comprehensive overviews, as well as promises and challenges, of these techniques when used *operando* on battery interphases are discussed in detail.

## 1 Introduction and Status of the Field

**Figure 1:**
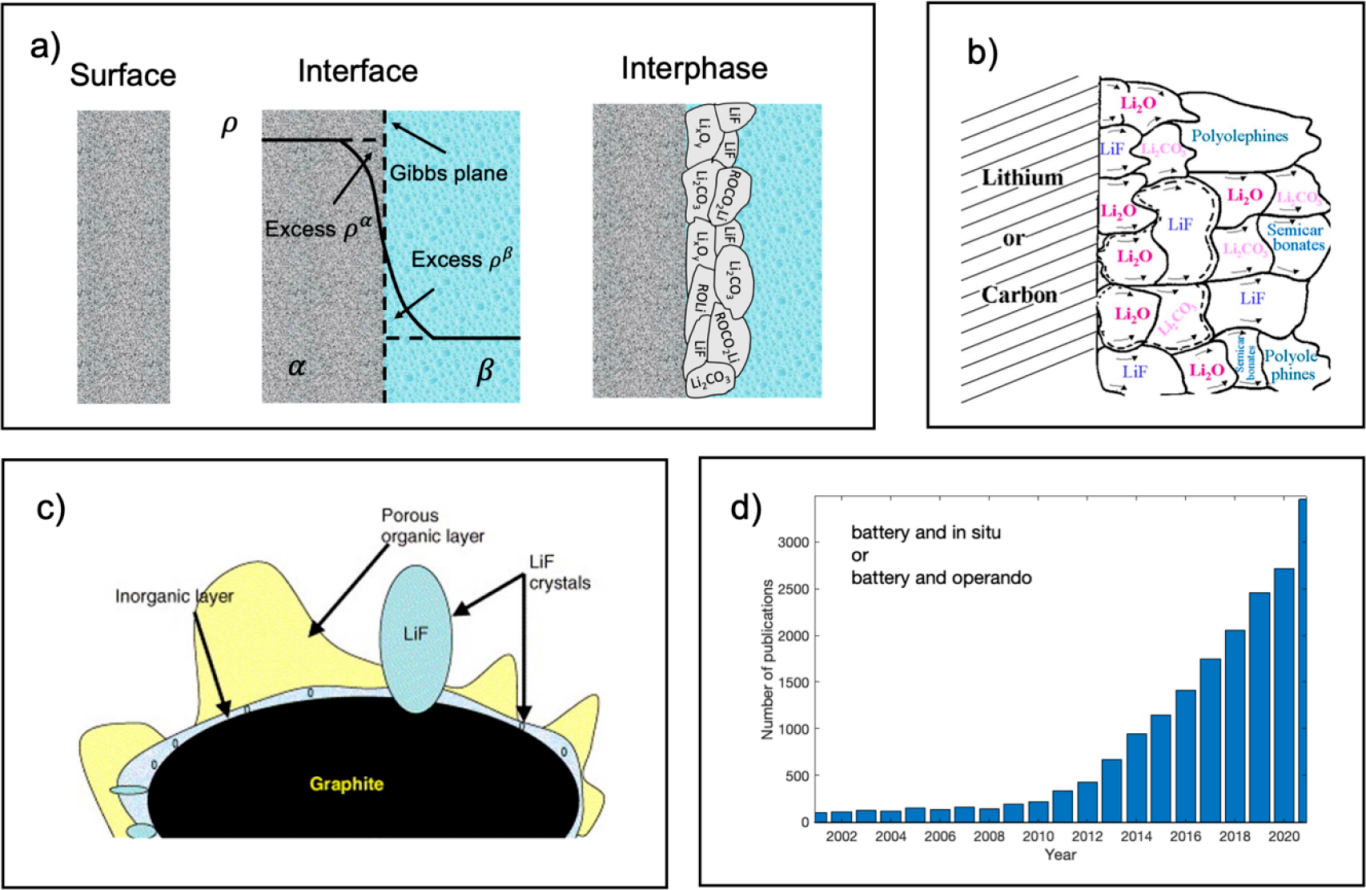
Schematic representations of (a) surface, interface, and interphase concepts. (b) SEI model by Peled et al., reprinted from ref (2) under CC-BY license. (c) SEI model by Edström et al., reprinted from ref (3) copyright 2005, with permission from Elsevier. (d) Number of publications per year with “battery and operando” or “battery and in situ” as part of title, abstract, or keyword, according to Web of Science, search date December 15th, 2022.

Understanding the electrode–electrolyte interactions is essential for battery development with interphases forming on both the anode and cathode during cycling. On the anode, the solid electrolyte interphase (SEI), which is an electronically insulating but ion conductive film, is formed during the first cycles and prevents continuous breakdown of electrolyte.^1^ The SEI (approximately 2 to 50 nm thick) consists of electrolyte decomposition products and is generally described as a bilayer structure: an inner denser and more inorganic layer (containing, e.g., LiF, Li_*x*_O_*y*_, Li_2_CO_3_) and an outer porous, more organic layer (e.g., lithium alkyl carbonates), as shown in Figure 1b–c.^2,3^ The cathode–electrolyte interphase (CEI) is also crucial but not as well understood. Similarly, to the SEI, the CEI consists of the electrolyte decomposition products on cathode, however it is yet unclear if it provides the same protection as the SEI or acts as a “solid electrolyte”.^4^

Before diving deeper into studying battery interphases, it is important to find a joint understanding of the often confusing concepts of surface, interface, and interphase. As shown on the Figure 1a, a **surface** is the outermost layer of a material or substance, whose physicochemical properties differ from those of the bulk, and is ideally referred to a surface in vacuum; however, it is also often referred to surface in contact with gas or liquid phase. The **interface**, however, is a 2D contact of two phases. According to the Gibbs convention, a transition between two phases α and β occurs in the region of finite thickness where the gradual changes of phase property ρ occur. The interfacial quantities of two phases *ρ*^*α*^ and *ρ*^*β*^ are considered as excess quantities compared with bulk phases α and β. While those changes occur gradually, it is possible to think of imaginary plane of separation, known as Gibbs plane.^5^ Electric charge excesses of two phases at the solid/liquid interface, known as electric double layer, is the most relevant interface encountered in the electrochemical systems.^6^ Applying the term interface implies that no reaction occurs between those two phases. On the other hand, an **interphase** is a three-dimensional contact of two phases, and unlike an interface, can consist of multiple phases and interfaces.^7^ The solid electrolyte interphase is the most relevant example of interphases encountered in batteries.^6^ As the definition of surface sensitivity varies between disciplines, in the current work we will refer to surface sensitive techniques as those capable to characterize battery interphases within their thickness range of 2 to 50 nm.^6^

Battery interphases are typically studied by applying *ex situ* techniques on individual electrodes before and after electrochemical cycling, e.g., X-ray photoelectron spectroscopy (XPS) or cryogenic transmission electron microscope (cryo-TEM). However, *ex situ* sample manipulation can influence the nature of the interphase (e.g., composition or thickness) and does not provide information about the intermediate reaction products and kinetics of the formation and growth. Therefore, it is essential to also perform these measurements with *operando* characterization, i.e., measurements performed during battery cycling, with measurement times significantly shorter than those of the battery charge–discharge cycle.

Interest in *operando* characterization of batteries has been increasing during the last 10 years (see Figure 1d), however most of *operando* techniques focus on bulk electrode or electrolyte properties rather than interphase characterization. For example, on December 15th, 2022, there were 22391 publication results which included “battery and *operando*” or “battery and *in situ*” in title, abstract, or keywords. A search within these 22391 publications revealed that 8411 publications included the keyword “cathode”, 9569 included the word “anode”, and 7154 included the word “electrolyte”. While “SEI or solid electrolyte interphase” was mentioned in a lesser number of 2045 publications, “CEI or cathode–electrolyte interphase” was the topic only in the 547 published reports. The most common technique was X-ray diffraction, (XRD) mentioned as a keyword in almost one-fourth of all reports. This quick literature survey confirms that only a small fraction of *operando* studies is focused on interphase characterization and that a larger academic effort toward development of surface sensitive *operando* techniques is necessary.

Here we provide a perspective on some techniques that have the required spatial and temporal resolution to probe interphases in batteries in *operando* mode. The focus is particularly on techniques that provide chemical, structural, and morphological information on the interphase on a representative electrode area: vibrational spectroscopy, X-ray photoelectron spectroscopy, neutron and X-ray reflectometry and grazing incidence X-ray scattering. Vibrational spectroscopy and XPS both provide information on chemical nature, but in XPS, the detected photoelectrons also contain electronic (interface) information. Reflectometry gives information on chemical nature, density, and the layered structure, while grazing incidence scattering can elucidate both morphology and structure of the interphase. While Raman and Infrared (IR) spectroscopy are bulk techniques, their surface sensitivity can be significantly enhanced; the mechanism and methods of this enhancement in *operando* configurations will be discussed in detail. On the other hand, XPS is a surface sensitive technique, but it is conventionally restricted to (ultra) high vacuum environments. We will therefore highlight the methodology of ambient pressure and liquid electrolyte environment XPS measurements. Reflectometry and grazing incidence scattering are intrinsically surface sensitive but are challenging to implement in *operando* configurations. A comprehensive overview of *operando* cell design and their relevance to battery systems will therefore be provided.^6,7^

## 2 Vibrational Spectroscopies: Raman, Fourier Transform Infrared (FTIR), and Sum Frequency Generation (SFG)

Vibrational spectroscopies are versatile and widely available techniques that are based on the light interaction with a material. They probe vibrational excitations in the sample which reflect, e.g., the structure, coordination, or chemical bonds in the sample. Raman and FTIR are undoubtedly the most widely known and used vibrational spectroscopy techniques in materials research generally and particularly in the energy storage field.^8^ While both Raman and FTIR are typically bulk techniques, their surface sensitivity can be triggered through surface enhancement in SERS (surface enhanced Raman spectroscopy) and SEIRAS (surface enhanced infrared absorption spectroscopy). Sum frequency generation spectroscopy is a less known vibrational spectroscopy technique that is based on two beams simultaneously interacting with the sample. SFG is intrinsically surface sensitive and uniquely suited for characterization of interfaces and interphases, however, the experimental setup and data analysis are rather complex.^9^

### 2.1 Raman Spectroscopy

Raman spectroscopy detects inelastically scattered light from material. For Raman spectroscopy, the probing depth is equal to the penetration depth δ and depends both on the laser wavelength and sample properties and is calculated according to eq 1:
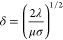
1where λ is the laser wavelength, μ is the magnetic permeability, and σ is the electronic conductivity.^12^ This equation shows that insulators and semiconductors have larger Raman penetration depths, while conductive materials have very small penetration depths. However, since only one in 10^7^ photons is scattered inelastically, the Raman signal is very weak when the probed sample volume is low. Therefore, for materials with a low penetration depth, the Raman signal will be very low or negligible. That means that Raman is not a surface sensitive technique, even though the penetration depth is low under certain conditions, and should be generally considered a bulk technique. For example, typical Li-ion cathode materials have penetration depths of a few tens to hundreds of nanometers.

In the battery field, Raman is mostly used for bulk material characterization and is suitable to analyze the core battery components anode, cathode, and electrolyte. The standard Raman surface sensitivity is not sufficient for battery interphase characterization. However, surface-enhanced Raman spectroscopy (SERS) gives an enhanced signal from molecules at the electrode surface and can probe the interphase on the electrode, even with the possibility to detect single molecules. This effect however is mainly observed on nanostructured surfaces of Au, Ag, and Cu, and is due to the electric field enhancement effect arising from the resonance with surface plasmons, which for the mentioned metals matches the laser wavelengths commonly used for Raman.^13,14^

**Figure 2:**
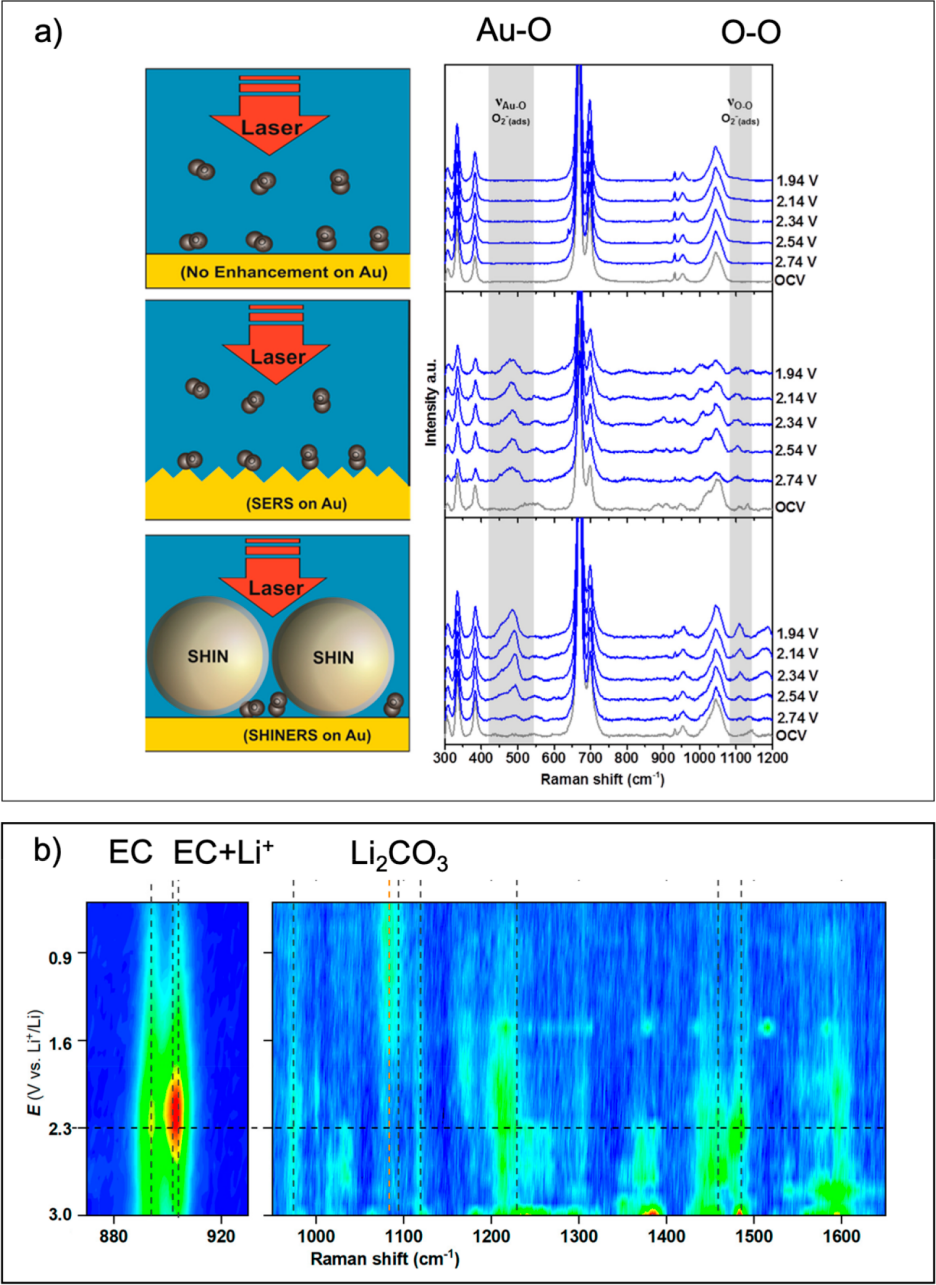
Raman spectroscopy for interphase characterization. (a) Left: Schematic representation of standard Raman (no enhancement), SERS, and SHINERS. Right: Corresponding *operando* spectra of Au/electrolyte interface, reprinted from ref (10) under CC-BY license. (b) Observation of electric double layer charging and SEI formation by *operando* SERS on Au substrate, reprinted from ref (11) under CC-BY license.

Given its exceptionally high surface sensitivity, SERS is ideally suited for probing electrode–electrolyte interfaces and interphases. To take advantage of SERS in *operando* battery environments, three set-ups could be used: shell-isolated nanoparticle-enhanced Raman spectroscopy (SHINERS), model systems using Au/Ag/Cu nanostructured substrates, or tip-enhanced Raman Spectroscopy (TERS), not covered in this perspective. Schematic representation of nonenhanced Raman, SERS with nanostructured substrate, and SHINERS on the top of a gold electrode, as well as corresponding *operando* Raman spectra of the gold/electrolyte interface, are shown in Figure 2a. It is evident that the enhancement effect from using a SERS substrate or SHINERS allows detecting Au–O and O–O species in the interphase, which are not captured by normal nonenhanced Raman.^10^

Mozhzhukhina et al.^11^ showcased a model study employing a SERS substrate by utilizing nanostructured gold in contact with the Li-ion battery electrolyte LP40 (1 M LiPF_6_ in 1:1 wt % ethylene carbonate (EC)/diethyl carbonate (DEC)) and recording Raman spectra during potentiostatic holds, Figure 2b. The obtained results provided information about the electric double layer charging, deduced from the ratio between EC molecules coordinating Li^+^ and free EC molecules, as well as the nature of early SEI formation, with the interphase mainly consisting of Li_2_CO_3_. This methodology provided powerful means to explore previously inaccessible information about interphase formation in battery relevant systems; however, the main disadvantage of the method is using model electrodes with a very high electrolyte/electrode ratio. Also, both the nature and morphology of the studied gold electrode surface are different from real battery composite electrodes which may affect the reaction kinetics. Particularly, the gold has electrocatalytic properties, therefore the results might not be easily transferred to commercial cells.

One way to study more realistic battery electrodes is using SHINERS. SHINERS are metal (typically Au) nanoparticles covered with a protective oxide layer that act as local electromagnetic enhancers on the probed sample. This method has been successfully used to investigate interphases in Li–O_2_ batteries (Figure 2a),^10^ and SEI formation in Li-ion batteries.^15^ The main advantage is that the nanoparticles could be mixed directly into or deposited on top of the composite battery electrode material. While at first glance, such an experimental setup seems like an easily adapted configuration, the main difficulties are finding a spot with the optimum enhancement and the low signal-to-noise ratio compared to using SERS substrates.^16^

### 2.2 FTIR

**Figure 3:**
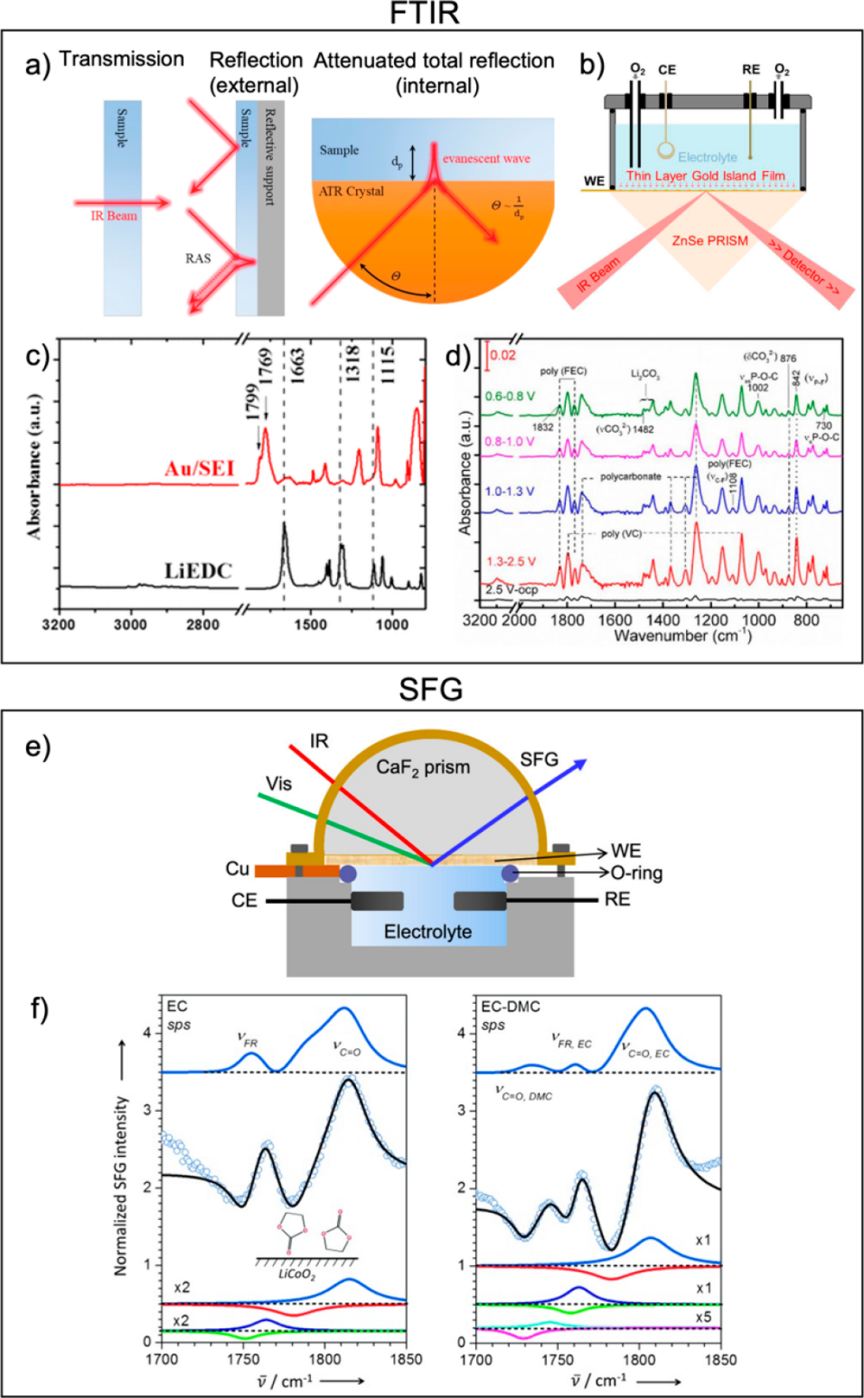
FTIR and SFG spectroscopies. (a) Schematics of different FTIR configurations: transmission, internal and external reflection, reprinted from ref (17) under CC-BY license. (b) Schematic representation of *operando* SEIRAS cell, reprinted from ref (18) under CC-BY license. (c) *Ex situ* ATR-FTIR spectra of LiEDC and SEI formed on the Au electrode, reprinted from ref (19) under CC-BY license. (d) *In situ* DRIFTS spectra of a 1 M LiPF_6_ in EC:EMC(1:2):VC (2 wt %) + FEC (10 wt %) electrolyte on Si-based electrodes during first lithiation, reprinted from ref (20) under CC-BY license. (e) Schematic representation of a SFG electrochemical cell, reprinted with permission from ref (21). Copyright 2020 American Chemical Society. (f) SFG spectra of the LiCoO_2_ surface in contact with EC and EC: DMC based electrolytes, reprinted with permission from ref (22). Copyright 2013 John Wiley and Sons.

FTIR is based on the absorption of infrared radiation by materials, and like Raman spectroscopy, it probes vibrational excitations. Since the vibrational modes are either Raman or IR-active due to molecular symmetries, the techniques are complementary.^23^ FTIR is a versatile technique, and measurements can be made in several different configurations (Figure 3a). Most commonly, experiments are performed in transmission mode, in which case the signal comes from the bulk of the studied phase. However, FTIR is also suitable for probing solid/liquid interfaces, where two main configurations are distinguished: internal and external reflection. In external reflection, the IR beam passes through the liquid phase before it is reflected from the electrode surface. In internal reflection measurements, the IR beam passes through the IR prism and a thin working electrode, which is typically directly deposited on the prism. A signal from the electrode/electrolyte interface is then obtained through the generated evanescent wave (Figure 3a–b). In both configurations, acquisition mode and signal processing must be carefully tuned to fully enhance the surface sensitivity of FTIR measurements.

Examples of external reflection FTIR applied to study electrode/electrolyte interfaces include subtractively normalized interfacial Fourier transform infrared spectroscopy (SNIFTIRS) and diffuse reflectance Fourier transform spectroscopy (DRIFTS). The SNIFTIRS technique involves signal processing in which the *operando* spectra are normalized with respect to a baseline spectrum. Using this approach, model interphases in Li–O_2_ batteries^24,25^ and interphases on Li-ion battery cathode materials^26−29^ have been investigated. However, the main disadvantage of the specular reflectance based FTIR is that it requires very smooth mirrorlike surfaces and consequently can only be applied to model systems. On the other hand, DRIFTS is based on diffuse rather than specular reflectance and therefore allows for a certain sample roughness and is suited to be employed with more realistic composite battery electrodes.^20,30,31^ For example, Yohannes et al. have used *in situ* DRIFTS to study the SEI evolution on Si-based electrodes in Li-ion electrolyte containing FEC and VC additives identifying numerous components of the interphase: organic phosphorus fluorides, polycarbonates, poly(VC), poly(FEC), Li_2_CO_3_, etc.,^20^Figure 3d.

Measurements in the internal reflection configuration normally employ attenuated total reflection (ATR) prisms, probing the interphase composition adjacent to the prism with the generated evanescent wave. In *operando* experiments, a thin electrode is typically either directly deposited on the prism or, alternatively, the electrode material is pressed against the prism. Clearly, the first setup is a model system, while the latter is a more realistic configuration. Several groups have used ATR-IR to study SEI formation,^32^ for example, Shi et al. identified the formation of lithium ethylene dicarbonate (LiEDC) on a Au electrode,^19^Figure 3c. In case of surface-enhanced infrared absorption spectroscopy (SEIRAS),^33^ gold islands are deposited on the ATR prism, which provides an enhanced interphase signal, Figure 3b. This method has been employed to determine Li–O_2_ battery reaction products.^18^ This technique is very powerful, but it is restricted entirely to model systems.

### 2.3 Sum Frequency Generation

Sum frequency generation is an intrinsically surface-sensitive nonlinear vibrational spectroscopy technique. It involves two beams, visible light, and tunable infrared light, which combine to produce an output beam of the summed frequency.^34^ The SFG process is only allowed in media without inversion symmetry, which is only true at the interfaces, and therefore, this technique only detects interfacial phenomena, avoiding any contribution from the bulk. SFG is a very powerful complementary technique to FTIR and Raman. *Operando* SFG experimental set-ups are very similar to FTIR and can also be performed either in internal or external reflection configuration. Schematic representation of an internal reflection SFG cell is shown in Figure 3e.

SFG experiments were explored by Yu et al. to demonstrate the preferential adsorption of EC molecules on a LiCoO_2_ surface in contact with an EC: DMC based electrolyte,^22^Figure 3f. SFG was also used to probe SEI formation on Au and Cu model electrodes detecting EC molecule reorientation and decomposition, SEI thickness variation upon cycling,^35^ and LiEDC as SEI component.^36^ Studies on Si anodes^37,38^ were performed utilizing either a Si single crystal or nanoparticles. The studies investigated the role of Si surface termination on the SEI composition and the role of CO formation on nano-Si. Additionally, they found that DEC reduces to epoxy moieties and showed voltage-dependent FEC reduction. The main drawback of SFG is that it is restricted to thin and smooth model surfaces (similar to FTIR), as well as a very complex experimental design and data analysis.

## 3 X-ray Photoelectron Spectroscopy

X-ray photoelectron spectroscopy (XPS) is a well-established tool for surface and interface analysis. XPS is inherently surface sensitive because photoelectrons can escape the sample only from the surface region without losing energy and thus specific chemical and electronic information. XPS information depth typically refers to the depth from which 95% of photoelectron signals are generated, which is around 5–10 nm by using Al Kα X-rays (1486.7 eV) to excite the photoelectrons.

While XPS is widely used *ex situ* in battery research, it is still challenging to use XPS on electrochemical devices or at least electrochemical interfaces under working conditions. When looking at solid state batteries or cells with a liquid electrolyte, the relevant interface, i.e., the contact between electrode and electrolyte, is sandwiched between two dense phases. Since photoelectrons have an inherently strong interaction with matter and thus a very short escape depth, on the order of a few to tens of nanometers, at least one of those phases needs to be very thin to access the interface. To further reduce interactions of the emitted photoelectrons on their way to the detector, XPS experiments are typically performed in UHV which makes such experiments incompatible with classical volatile liquid battery electrolytes. With (near) ambient pressure XPS (APXPS), the latter vacuum constraints are relieved allowing up to tens or even hundreds of mbar gas pressure in the analysis chamber, so that liquid battery electrolytes can be maintained during the experiment.^39^ In this perspective, we focus on *operando* characterization of the solid/liquid interface using APXPS. For a general overview of how photoelectron spectroscopy has contributed to our understanding of interphases in batteries and how *operando* experiments can be achieved in solid-state batteries, we refer to, e.g., refs (40−42).

**Figure 4:**
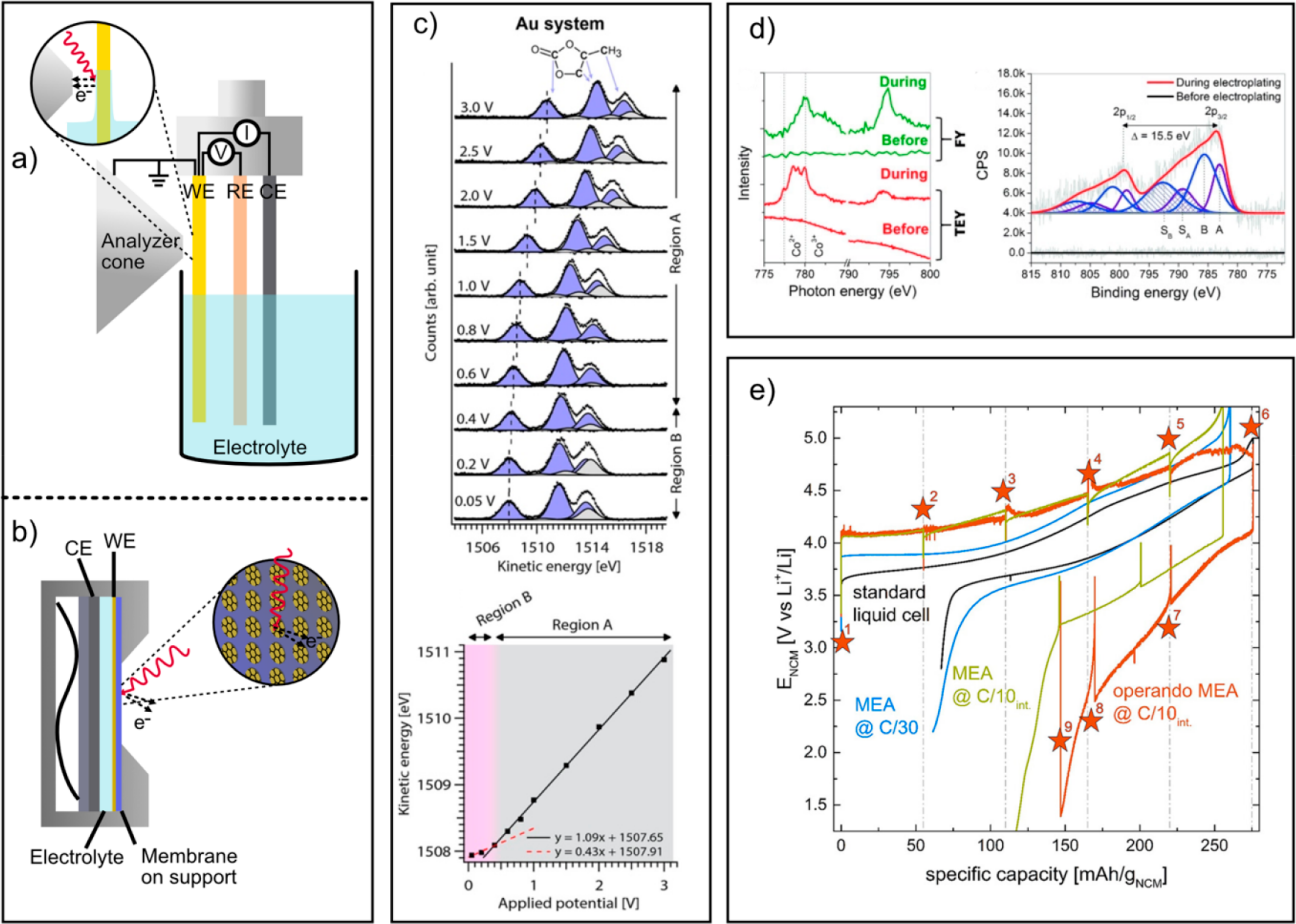
Schematics of *operando* APXPS configurations: (a) dip-and-pull setup and (b) sealed cell setup. (c) Top: C 1s APXPS data recorded using a dip-and-pull setup based on a Au electrode in 1 M LiClO_4_ in PC electrolyte where the applied voltage was lowered stepwise from OCV 0.05 V vs Li/Li^+^. Bottom: Changes in carbonate peak position vs applied voltage indicating different interfacial charge transfer behavior (reproduced from ref (48) according to the terms of the CC-BY 4.0 license). (d) *Operando* X-ray absorption (left) and APXPS (right) data of Co electrodeposition on graphene (from 4 mM CoSO_4_ in H_2_O) recorded using a sealed membrane cell (reproduced with permission from ref (52). Copyright 2015 Wiley-VCH Verlag GmbH &Co. KGaA, Weinheim). (e) Electrochemistry data of a semisealed cell called membrane electrode assembly (MEA) for *operando* APXPS in comparison to standard laboratory liquid electrolyte cell (reproduced from ref (57) according to the terms of the CC-BY 4.0 license).

While the general concept of APXPS has been around for several decades^43,44^ the geometries have changed, and technological advances in analyzer design using several differential pumping stages and electron focusing lenses^45,46^ led to more widespread application for electrochemical interfaces. Thus, one could move away from the early experiments using a confined liquid jet through the vacuum chamber and introduce more elaborate experimental set-ups into the analysis chamber such as electrochemical cells. The two currently pursued approaches are based on either an open beaker-type electrochemical cell placed directly into the analysis chamber or a membrane-sealed closed electrochemical cell. In the former, the entire analysis chamber operates at elevated pressures in the range of a few to tens of millibar (typically around the electrolyte solvent vapor pressure), and the liquid can be probed directly. Electrochemical set-ups in this open configuration are based on the dip-and-pull approach^47,48^ (illustrated in Figure 4a) or a titled sample approach.^49,50^ In the latter sealed cell configuration, the liquid and elevated pressure environment are separated from the main analysis chamber by a solid, thin, electron transparent membrane based on, e.g., graphene,^51,52^ graphene oxide,^53^ or nonstoichiometric silicon nitride.^54^ Both configurations are illustrated in Figure 4a–b together with spectroscopic and electrochemical data exemplifying the capabilities of either experimental setup (Figure 4c–d). Figure 4c shows in the top the characteristic C 1s spectra for a 1 M LiClO_4_ electrolyte meniscus on a gold electrode that undergoes stepwise potential changes from the OCV to 0.05 V vs Li/Li^+^. In Figure 4c bottom, the XPS peak shifts in kinetic energy are plotted vs the applied voltage, and from the changing slope we can derive insights about interfacial charge transfer. The data in Figure 4d show successful electroplating of Co on graphene during *operando* X-ray adsorption (left) and XPS (right) measurements. This example is only indirectly related to battery research as the membrane electrochemical cells are to the best of our knowledge not yet used in dedicated battery research. These data are thus intended to exemplify APXPS capabilities to follow *operando* electrodeposition of metals that could be transferred to battery research, i.e., to *in situ* deposit a metal anode on the graphene membrane and study its interaction with the electrolyte.

Generally, APXPS has gained increasing attention as a tool for battery studies, as it allows for the analysis of samples at near-ambient pressure rather than in vacuum. The technique has been used to study a range of battery materials, including lithium-ion batteries,^55−58^ sodium-based batteries,^59^ and solid-state batteries^60^ In these studies, APXPS has been used to investigate a variety of phenomena, such as the surface chemistry of electrodes and electrolytes,^39,50,61^ the evolution of reaction products during charge and discharge^60^ and degradation mechanisms,^57^ as well as interfacial charge transfer phenomena.^48,58,62^

One key advantage of APXPS is that it allows for the analysis of batteries under conditions that are more representative of their actual operating environment. Early work using APXPS on a cycled electrode without any pretreatment before the measurement (no washing or drying of the electrode) showed that the SEI studied in post-mortem experiments is representative for the formed SEI.^55^ However, this does not answer the open questions about how the SEI builds up during cycling and which intermediates are crucial in the process. To answer these questions, *operando* measurements, e.g., using the above-described electrochemical set-ups inside a spectrometer are required. However, these experiments are demanding, and one can question how closely we can mimic battery operating conditions and how realistic *operando* APXPS battery studies can be. So far, only thin film and densely calendared simplified electrodes have been used to avoid wetting the entire electrode with a liquid film much thicker than the APXPS probing depth due to capillary forces. Will we be able to use more realistic electrodes in the future to study the influence of porosity, binder, and conductive additives on the interface reactions and formed interphases?

The current experimental set-ups use a vast excess of electrolyte compared to commercial cells of any format. It might be easier to approach more realistic electrode/electrolyte ratios in sealed membrane cells, but this geometry limits the studies to the “back side” of the electrode or individual particles, again leaving the question of how representative this limited probing volume is for an entire battery. It might be possible to use a dip-and-pull approach in a different configuration to reduce electrolyte excess, but connecting the liquid electrolyte meniscus to the bulk electrolyte is vital to a functioning electrochemical cell. Here, the question arises if the thin liquid layer is representative of a bulk battery electrolyte in terms of composition and, for example, conductivity/ion transport. According to previous work, both electrolyte composition^48,61^ and ion transport in APXPS^64^ can be questioned or need at least careful consideration during experimental planning to be able to correlate the results to realistic battery applications. An alternative approach could be the tilt-trough cell in which a sample and trough, or beaker, are tilted relative to the horizontal liquid electrolyte surface using a vertical analyzer geometry as described in.^63^ However, also in this configuration, electrode wetting, finding a spot where the liquid film is thin enough to probe through to the solid with the given excitation energy, and a nonideal electrode orientation with respect to each other can impact the *operando* results. Concerning the electrolyte composition, another challenge arises as typical battery electrolytes are multicomponent systems consisting of several solvents, at least one salt, and additives. At present, successful APXPS results are reported only for simplified electrolyte formulations consisting of a single solvent and a single salt. The dip-and-pull setup allows for realistic concentrations around 1 M but keeping electrolytes with multiple solvents with different vapor pressures stable inside the measurement chamber for the duration of the experiment is still a challenge.

Compared to a battery or standard three-electrode configuration, the electrochemical setup in *operando* APXPS measurements is more complex as it also includes the photoelectron analyzer. The working electrode, i.e., the sample, is typically grounded with the photoelectron analyzer (Figure 4a) to maintain a reference in photoelectron binding energy. Voltage changes in the electrochemical cell will therefore not be reflected in photoelectron peak shifts of working electrode species but in XPS peak shifts for the electrolyte species (Figure 4c). It is, therefore, crucial to perform the experiments in a three-electrode setup to capture the true electrochemical response of the system. Choosing an appropriate reference electrode is an essential step in electrochemical studies^64^ but even more important when exploiting XPS capabilities to derive electronic interface information from binding energy shifts. It is also critical to consider the choice of counter electrode to avoid crosstalk interactions between the working and counter electrode, especially in post-Li systems where the metals (such as Na, K, Ca) are highly reactive with the electrolyte and do not form a stable SEI leading to continuous side reactions and increased polarization. Finally, the changed geometries for the dedicated *operando* cells lead to “electrochemical” artifacts such as increased polarization as shown in Figure 4e for a semisealed cell called membrane electrode assembly (MEA) for *operando* spectroelectrochemistry in comparison to a standard laboratory liquid electrolyte cell.^57^

From post-mortem studies, it is well-known that cycled battery electrodes with an SEI can be sensitive to X-ray illumination. Especially the electrolyte salts seem to degrade during XPS characterization.^65,66^ In APXPS the liquid electrolytes have also displayed degradation under X-ray illumination. While the pure solvent itself was stable for around 45 min of continuous illumination, an electrolyte based on the same solvent but with a conductive salt showed severe degradation during roughly the same time of X-ray exposure. However, it was noted that changing measurement spot on static drops or during dip-and-pull experiments yielded “fresh” electrolyte surface with no detectable signs of radiation damage.^39^ This implies that the electrolyte decomposition seems to be localized to the irradiated area, and the diffusion length of the decomposition species is limited. Here, the large electrolyte volume in the beaker during a dip-and-pull experiment could even be beneficial, as decomposition products get easily diluted in the vast liquid excess.

Finally, data interpretation should be considered as it is already not straightforward in post-mortem interphase studies. For cycled battery electrodes, peak shifts occur due to changes in the electrode potential, due to redox reactions, double layer, and SEI formation, just to name a few effects.^67−69^ The added liquid electrolyte and driving electrochemical reactions inside the spectrometer further complicate the photoelectron spectra. As XPS typically probes an atom’s nearest chemical environment, it becomes challenging to distinguish, for example, the carbonate electrolyte solvent and the carbonate decomposition product.

## 4 Neutron and X-ray Reflectometry and Off-Specular Scattering

**Figure 5:**
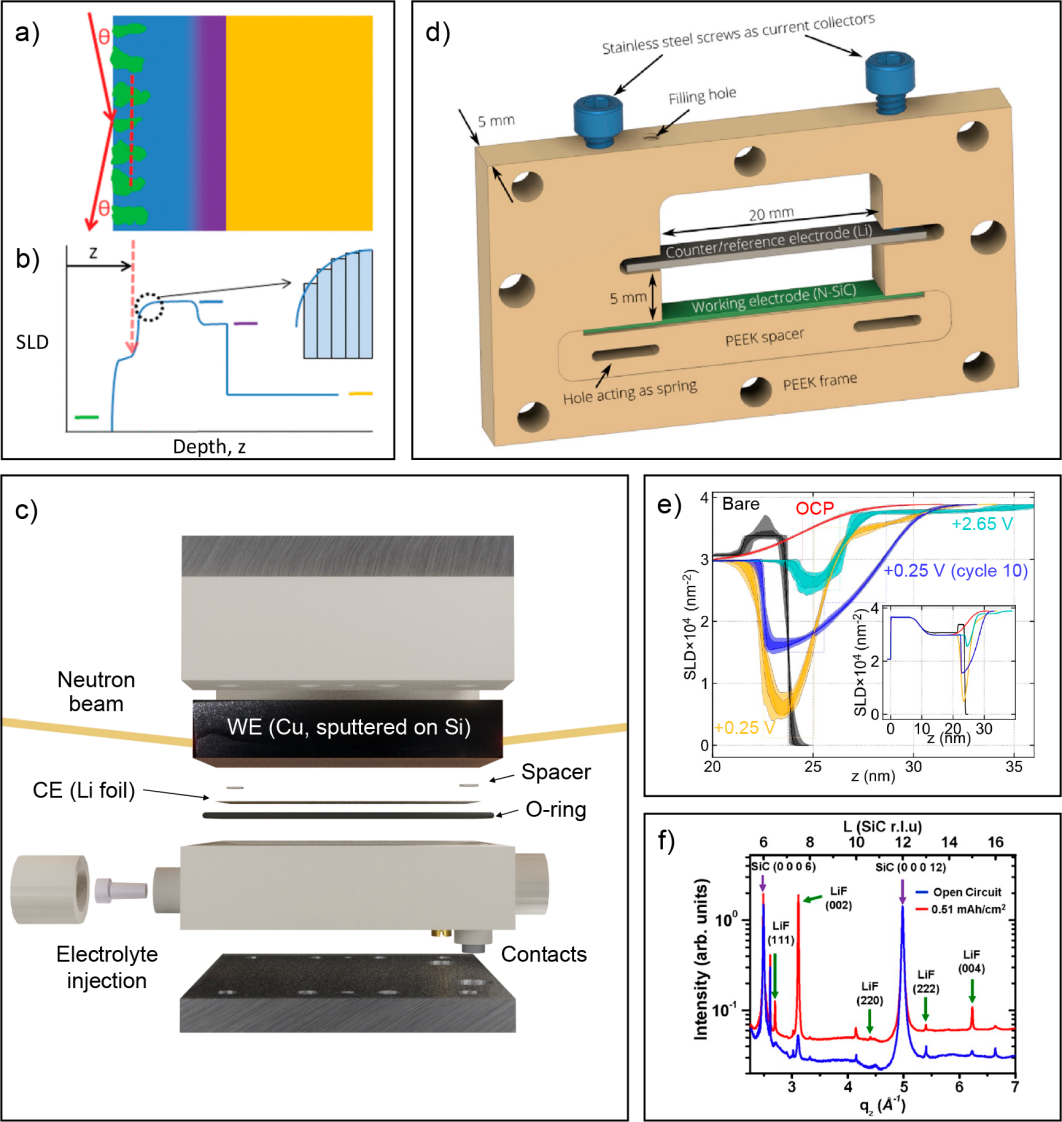
Neutron and X-ray reflectometry and off-specular scattering. (a) Schematic of a sample with a layered structure containing in-plane inhomogeneities; the dashed line shows the coherence length of the beam. (b) The corresponding average SLD-profile. Note that the colored bars show the bulk SLD of each material present in panel a. Reprinted (adapted) with permission from (80). Copyright 2012 American Chemical Society. (c) Neutron reflectometry cell for studying the SEI formation on Cu. (d) X-ray reflectometry cell. Reprinted (adapted) with permission from ref (79). Copyright 2021 American Chemical Society. (e) Neutron-SLD profiles obtained from a reflectometry experiment on a W-electrode swept between 0.25 and 2.65 V in 1 M LiPF_6_ in EC:DEC. Reprinted (adapted) with permission from ref (73). Copyright 2019 American Chemical Society. (f) GIXRD curves recorded at open circuit potential and after 0.51 mAh/cm^2^ lithiation. Reprinted (adapted) with permission from ref (81). Copyright 2012 American Chemical Society.

Reflectometry is a technique often used for thin film characterization. In the most common type of reflectivity experiment, the sample is illuminated with a beam at grazing incidence, and the intensity of the specular reflection is determined as a function of the momentum transfer vector, *Q⃗*. The momentum transfer vector can be varied by changing the wavelength, λ, or incidence angle, θ, according to
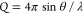
2This yields information on the scattering length density (SLD) across the sample interphase. The SLD is determined by the composition and density of a material through eq 3.
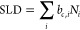
3where *b*_*c*__,*i*_ and *N*_*i*_ are the coherent scattering length and number density of an element *i*. The scattering length describes the scattering power of an element; for X-rays, this parameter is proportional to the number of electrons, while each isotope has a different neutron scattering length. At any interface in a sample where the SLD changes, the beam will be refracted. The reflections from different interfaces inside the sample will travel different path lengths to reach the detector, and thus, the interference between these different reflections encodes information about the layered structure of the sample. To extract this information, a model for the SLD as a function of the sample depth, (Figure 5a–b) can be extracted by fitting the reflectivity data.

Samples with a layered structure, wherein the layer thicknesses range from below one to hundreds of nanometers can be studied using X-ray and neutron reflectometry.^70,71^ The measurements probe the averaged sample structure along the surface normal. More precisely, a sample with in-plane inhomogeneities smaller than the coherence length of the beam, see Figure 5a (the dashed line denotes the coherence length of the probe), will be indistinguishable in a reflectometry experiment from a homogeneous sample with the same SLD-depth profile (Figure 5b). Samples with in-plane inhomogeneities on length scales larger than the coherence length of the beam should be avoided as it is equivalent to measuring on multiple different samples simultaneously, vastly complicating data analysis.^71^ Additionally, successful reflectometry experiments require electrodes with very low surface roughness, typically to be below 2 nm.^71^ Consequently, reflectometry experiments need to be conducted on 2D model systems mimicking the electrode surfaces of interest. Such electrodes can be prepared by depositing the active material on a smooth substrate, ideally atomically flat, using techniques like pulsed laser deposition^72^ or magnetron sputtering.^73^

Depending on whether an X-ray reflectometry (XRR) or neutron reflectometry (NR) experiment is to be carried out, the cell design considerations differ significantly (exemplified in Figure 5c–d). Neutron beams in reflectometers typically have large “footprints”, requiring NR cells to be designed with large electrodes to maximize the reflected intensity and minimize measurement times. The active electrode areas often range around 20–40 cm^2^_._^74,75^ A neutron beam can pass through these large cells without excessive attenuation as it typically enters the cell through a single crystal of Si^74−76^ or quartz block^77,78^ (in which the beam experiences minimal attenuation). The electrode material is sputtered on top of this neutron transparent substrate in a thin layer. In an X-ray experiment, on the other hand, the beam enters the cell through an X-ray transparent window (for instance, Kapton as in Figure 5d) and passes through the electrolyte before it reaches the interface of interest. Since X-ray beams usually have a smaller footprint, but are more easily attenuated, XRR cells are designed with smaller active electrode areas than NR cells, commonly on the order of 1 cm^2^.^72,79^ In both cases, the cells are generally designed with a large excess of electrolyte and without conventional separators to enable a more straightforward analysis.^67,74,74^

Most reflectometry measurements are carried out *in situ*, but measurement times can be optimized to allow *operando* characterization, as well. A higher beam flux will decrease the measurement time, which is why XRR measurement can often be acquired within a couple of minutes,^79^ whereas a NR-curve typically requires hours to obtain.^74^ However, this time can be significantly reduced by restricting the Q-range probed during each scan, even allowing NR measurements to be carried out *operando* with acquisition times of a few minutes.^76^ The reflected intensity quickly falls off with higher Q-values, leading to poor signal-to-noise ratios, requiring long measurement times. By excluding the higher Q-values, measurements are faster with the drawback of losing information, especially about thin layers, being lost. Other ways to decrease the measurement time include improvements in instrumentation, such as the focusing optics introduced at the beamline Apparatus for Multi Option Reflectometry (AMOR) at Paul Scherrer Institute (PSI), which can both help decrease the measurement times and allow smaller samples to be used.^82^

Both XRR and NR are often termed nondestructive probes. However, beam damage is still an important issue to keep in mind for the design of accurate XRR experiments. Unfortunately beam damage remains widely underreported issue.^83^ For example, it was demonstrated that X-ray beam induced significant morphological changes in sulfur cathodes in Li–S batteries.^84,85^ The effects of X-ray induced sample damage can for instance be minimized by regularly moving the beam to new points on the sample over long experiments.^79,86^ Due to the much weaker interactions between neutrons and the sample compared to the photons generated in a synchrotron, sample damage is usually not considered as a problem in neutron experiments.^87^

The use of reflectometry and (X-ray) surface scattering to study electrode surfaces was pioneered in the late 1980s and 1990s, studying mostly single crystalline substrates and phenomena such as underpotential deposition of metal mono/bilayers and liquid ordering at the electrode surface.^88−93^ The reflectometry studies on battery electrode interphases have so far focused on lithium-based electrodes. Probably due to their large practical importance and the excellent sensitivity for changes in lithium concentration at the electrode/electrolyte interface in NR measurements (due to the negative scattering length of Li). For this reason, a large body of work exists also on lithiation of anode materials, foremost Si (which is also almost neutron transparent and readily manufactured with appropriate surface roughness), using neutron reflectometry.^94,95^ Additionally, reflectometry has been used to study SEI layers on both carbon^76^ and metals with low reactivity like Cu^74^ and W,^73,96^ which can readily be sputtered on a substrate. Further, the CEI on the cathode materials LFP,^97^ LMNO,^98^ and LCO^99^ have also been investigated. The selection of materials to study using reflectometry is limited by which combination of materials (substrate, electrode, electrolyte) gives a good enough contrast for the formed interphase and which electrode materials can be prepared as thin, smooth films. Albeit more challenging, it should also be possible to study more reactive electrode materials like lithium metal, but this would require more advanced experimental facilities where, for instance, sputtering equipment and cell assembly glove boxes are onsite and connected via inert transfer shuttles or located in a dry room to avoid exposure to moisture as sputtered electrodes are transferred into the glovebox.

The information about the SEI cannot be directly inferred from the measured data. Instead, the data need to be fitted with a model for the SLD profile through the sample. However, a reflectometry curve is not necessarily uniquely fitted by one model of the SLD profile, requiring careful and well-motivated model selection. Further, when electrochemical interphases are studied, it is often difficult to tell a priori which layered structure would best represent the electrode interphase. To systematically select models with the appropriate complexity level, Dura and co-workers have proposed using the Bayesian information selection criterion.^100^ Ideally, to maximize the information that can be obtained in an experiment, all layers at the electrode interphase would have the same SLD except for the layer(s) of interest.^73^ It has been demonstrated that careful selection of electrode material and deuteration of the electrolyte to approach this situation can be useful and important tools to optimize contrast for the solid electrolyte interphase in neutron reflectometry measurements. This way, more complex models of the electrode interphase can be reliably fitted to the data, allowing us to better understand the structure of the SEI.^68^Figure 5e exemplifies how combining a tungsten electrode on a Si substrate in combination with a deuterated electrolyte provided good contrast for the electrode/electrolyte interface, revealing a two-layer structure of the SEI.^73^ Another strategy to guide the fitting of neutron spectra and gather more information on the interphase is to do multiple measurements with different levels of electrolyte deuteration.^101^ To advance the understanding of battery interphases through reflectometry measurements, contrast optimization is one of the most important aspects.

To add to the information on battery interphases obtained from specular reflectometry, *in situ* measurements with techniques more sensitive to in-plane inhomogeneities could be a powerful complement. This could, for instance, be off-specular reflectometry, which can be obtained simultaneously with the specular data at many instruments. Another option that we believe is worth exploring for interphase studies is grazing incidence small angle X-ray scattering (GISAXS) and grazing incidence X-ray diffraction (GIXRD).

At the nanoscale, grazing incidence scattering methods can provide extensive information about particle morphology (size and shape) and structure (grain size and lattice characteristics). Examples of *operando* GISAXS in the battery field include studies on the evolution of the metal oxide electrode mesostructure during cycling,^102^ as well as lithium metal nucleation and growth.^103^ There are very few reports of SEI characterization by GISAXS or GIXRD. While the SEI is a film, its structure in fact resembles a mosaic with nanometer scale crystals or amorphous domains, which makes it suitable to be studied by GISAXS/GIXRD. For a typical Li-ion battery electrolyte, it is expected to have a good contrast in the experiment with a scattering length density (SLD) estimated to be 1.4 × 10^–5^ Å^–2^ for the 1 M LiPF_6_ in ethylene carbonate (EC) and diethyl carbonate (DEC) solvents mixture, while the SLDs of the inorganic SEI layer components are 2.1 × 10^–5^ for LiF, 1.8 × 10^–5^ for Li_2_CO_3_, and 1.6 × 10^–6^ Å^–2^ for Li_2_O (considering the wavelength of 1.54 Å). The outer organic SEI layer is expected to have a similar SLD as the electrolyte leading to low contrast in the measurement. Therefore, mainly, the formation and evolution of the inner, more inorganic SEI layer will be probed by *in situ*/*operando* grazing incidence techniques. For example, Chattopadhyay et al. have found that LiF was the main crystalline SEI product formed on epitaxial graphene detected by *in situ* grazing incidence XRD^81^ (Figure 5f).^81^ Like reflectometry experiments, model systems with extremely smooth electrode surfaces need to be studied. Therefore, it is critical that such experimental data is used hand in hand with atomistic and multiscale computational models targeting to link the interfaces and interphases properties to battery performance. Since SEI formation is a dynamic process, high temporal resolution is required (1 min or less) in order to monitor its nucleation and growth kinetics. To meet these requirements, a high signal-to-noise ratio, high temporal resolution, and simultaneous GISAXS/GIXRD are needed which can only be offered at the large-scale facilities.

## 5 Future Field Development: Opportunities and Challenges

*Operando* studies on batteries have already provided further interphase insights into otherwise not directly observable processes like electrochemical potential distribution and early SEI formation (e.g., using SERS^11^ and APXPS^48^). However, most of the techniques capable of the *operando* characterization of interphases in batteries involve model systems. For example, grazing incidence and reflectometry methods require extremely smooth metal surfaces, whereas surface-enhanced Raman and SEIRAS rely on the use of very particular nanoscale morphology substrates. The *operando* setup also often requires compromises in the electrochemical cell design as, for example, in APXPS where electrolyte volume and electrode arrangement differ significantly from lab scale test batteries. Despite their model electrochemical set-ups, these techniques still provide much needed information on the nature and properties of battery interphases. *Operando* surface sensitive techniques for batteries have the potential to provide: (1) better understanding of how the electrode surface chemistry and interphase structure evolve during charging and discharging, (2) identification of reaction products and intermediates formed during battery operation, and (3) testing the validity of results obtained through *ex situ* measurements.

Thus, *operando* surface sensitive techniques provide key insights to identify the degradation mechanisms and to guide design strategies to improve the battery lifetime. In this Perspective, we have highlighted some of the examples where *operando* experiments have pushed battery interphase understanding. In the following, we would like to raise awareness of some open research questions to inspire future work to further increase the impact and relevance of *operando* interphase studies:

- **Electrode composition and porosity.** Most of the covered techniques require thin film or nonporous model electrodes, which inherently have different properties as compared to composite battery electrodes. One exception being SERS where adding SHINERS to composite electrodes represents one way to maintain electrode morphologies. For other techniques where electrode porosity is an issue, densely calendared electrodes or sputtered thin films could be a way toward more realistic systems.
- **Electrolyte volume and composition.** Most of the *operando* cells are overflooded systems often without a separator, which combined with low surface area electrodes, results in very high electrolyte/electrode ratios. This in turn can results in different effects electrolyte additives or impurities have on the cell performance. In order to improve data reproducibility and reliability, we encourage to accurately calculate, and report employed electrolyte/electrode ratios. Also, electrolyte composition is often determined by the employed techniques: Vibrational spectroscopy and XPS require a smaller number of different components in order to get meaningful species deconvolution and neutron-based techniques benefit from electrolyte deuteration. While a decreased or simplified electrolyte composition can provide better insight on the interfacial processes and easier comparison with computational data, one should also be aware of possible synergies between different components in realistic but complex electrolyte systems.
- **Electrochemical setup.** Typically, advanced *operando* characterization cells have very complex geometries and lacking stack pressure, both resulting in high(er) internal resistance. A high ohmic drop, particularly when using a two-electrode configuration, will result in significant distorted electrochemical curves as compared to battery cycling in coin or pouch cell configuration. We encourage to benchmark *operando* electrochemical setups with laboratory test batteries in terms of electrochemical response. In terms of setup design, efforts should be made to decrease internal cell resistance and employ three-electrode configurations. This is particularly relevant for APXPS, since working the electrode is grounded with analyzer, and the use of reliable reference electrode is a necessary requirement.
- **Beam damage**. Beam damage is very important, but largely neglected and underreported,^83^ issue for the synchrotron X-ray techniques, particularly when studying interphases. While this is less of a problem with vibrational spectroscopy, high Raman laser power can also result in sample burning.^104^ Therefore, it is important to disentangle electrochemically induced and beam-induced changes and to monitor possible signal changes due to illumination at open-circuit potential, reproduce experiments on different measurement position on the electrode and replenish electrolyte when possible. Beam damage can be either assessed visually, e.g., bubbles formation in electrolyte, laser-induced spots on the sample surface, change in particle dimensions, or by a signal change not representative to the electrochemical procedure (e.g., significant signal change at open-circuit potential). For the better data reproducibility and reliability, it is recommended to assess and report dose limits for performed experiments, expressed in Grays (energy absorbed by kg) or incidence flux density (photons/cm^2^), as well as methodologies to determine the beam damage.^83^ Some ways to reduce the beam damage involve using higher energy and lower doses X-rays and carefully optimizing sampling time.^83−85,105^ Notably, beam damage is chemistry-specific problem;^83,105^ however, chemical reactions occurring during the beam damage are not thoroughly investigated and more efforts are needed toward understanding the mechanisms of beam damage and possible ways for its mitigation.
- **Data interpretation.** Data interpretation presents very different challenges depending on the technique. For the techniques that provide chemical information, such as XPS and vibrational spectroscopy, the main challenge is species assignment, spectral deconstruction, and fitting in a complex multicomponent environment. For scattering and reflectometry, the main challenge is finding appropriate and coherent models for data fitting. To achieve the most reliable data interpretation, it is essential to combine different characterization techniques and computational studies, and to adhere to FAIR data principles.^106^

In general, to translate the results of *operando* model studies to real-life batteries, the limitations of the model systems need to be explored to determine whether the results can be extrapolated to more realistic conditions. Additionally, experimental and simulated data from multiple sources should be combined to get a more complete picture of the behavior of the battery^107^ confirming the relevance of the results. Therefore, the development and correct data interpretation of surface sensitive *operando* techniques are not only one of the most significant challenges but also a great opportunity for future battery research.
